# Unbiased Quantitative Proteomics Reveals a Crucial Role of the Allergen Context for the Activation of Human Dendritic Cells

**DOI:** 10.1038/s41598-017-16726-2

**Published:** 2017-11-30

**Authors:** L. Strasser, H.-H. Dang, H. Schwarz, C. Asam, F. Ferreira, J. Horejs-Hoeck, C. G. Huber

**Affiliations:** 1 Department of Molecular Biology, Division of Chemistry and Bioanalytics, University of Salzburg, Hellbrunner Straße 34, 5020 Salzburg, Austria; 2 Department of Molecular Biology, Division of Allergy and Immunology, University of Salzburg, Hellbrunner Straße 34, 5020 Salzburg, Austria

## Abstract

Worldwide, more than 1 billion people suffer from allergic diseases. However, until now it is not fully understood how certain proteins can induce allergic immune responses, while others cannot. Studies suggest that allergenicity is a process not only determined by properties of the allergen itself but also by costimulatory factors, that are not classically associated with allergic reactions. To investigate the allergenicity of the major birch pollen allergen Bet v 1 and the impact of adjuvants associated with pollen, *e.g*. lipopolysaccharide (LPS), we performed quantitative proteome analysis to study the activation of monocyte-derived dendritic cells (moDCs). Thus, we treated cells with birch pollen extract (BPE), recombinant Bet v 1, and LPS followed by proteomic profiling *via* high-performance liquid chromatography and tandem mass spectrometry (HPLC-MS/MS) using isobaric labelling. Enrichment and pathway analysis revealed the influence of regulated proteins especially in cytokine signalling and dendritic cell activation. We found highly regulated, but differentially expressed proteins after treatment with BPE and LPS, whereas the cellular response to Bet v 1 was limited. Our findings lead to the conclusion that Bet v 1 needs a specific “allergen context” involving cofactors apart from LPS to induce an immune response in human moDCs.

## Introduction

Allergies are widespread disorders which currently affect up to 30% of the population in European countries. As a consequence, their socioeconomic impact considering costs of health care, influence on work and education, or quality of life is constantly increasing^1,2^. In the northern part of Europe, birch pollen is a central source of allergens^3^. Ninety-five percent of patients suffering from birch pollen allergy are sensitized to the major birch pollen allergen, Bet v 1^3,4^, which makes it a major target for allergy diagnosis and therapy^5^. Currently, pollen extracts are applied to allergic patients in a sublingual or subcutaneous manner for allergen-specific immunotherapy (SIT). Thereby a tolerance state should be induced by modulating the patient’s T and B cell response. However, these natural extracts generally show inconsistent composition, while also containing non-allergenic compounds. Thus, therapy often takes a long time and can induce local or systemic inflammatory responses in patients. The use of recombinant allergens could provide better-defined and highly-purified products for immunotherapy preventing these potential side effects. Nonetheless, when comparing birch pollen extracts with recombinant Bet v 1 for immunotherapy, a lower efficacy was observed for the recombinant product^6,7^.

How certain proteins can cause allergic sensitization in some individuals while not in others is still an open question, even if the process of allergic sensitization is quite well understood on a cellular level. In particular, dendritic cells (DCs) are crucial players in the initiation phase of an allergic immune response. As antigen presenting cells (APCs), they promote T cell differentiation by using a series of three signals. First, they induce antigen-specific stimulation of T cells by presenting antigenic peptides *via* their major histocompatibility complex class II (MHC-II) proteins. Secondly, costimulatory interaction of receptor molecules on DCs (*e.g*. CD80 and CD86) with their respective ligands on T cells (*e.g*. CD28)^8^ are required. In the end, the secretion of specific cytokines which are in most cases produced by activated DCs induces the polarization of naïve T cells into effector T cells^9–12^. In general, an allergic immune response is characterized by the induction of a CD4^+^ T_H_2 phenotype^13–16^. What remains unclear is the detailed molecular mechanism underlying the activation of the T_H_2 program. Until now IL-4 is assumed to be a key signal for T_H_2 polarization. Even though the cellular source for IL-4, which is required to initiate the T_H_2 program, is still under debate^17^ and DCs are probably not the main producers of IL-4, their importance for a T_H_2 response was reported in several studies as reviewed by Na *et al*.^9^.

An important factor seems to be the individual allergenicity of a protein, which describes the property of an antigen to cause an allergic immune response^18^. Allergens have been clustered into structural groups which consider characteristics such as IgE-binding epitopes, molecular mass, and proteolytic stability^19^. However, this clustering does not facilitate an estimation of allergenicity, and therefore, allergenicity seems to be a multifactorial process. It depends not only on structural and biochemical aspects of the allergen but also on genetic predisposition and the exposure to cofactors^18–22^. Pivotal pollen-derived cofactors can for instance be pollen associated lipid mediators, capable of inhibiting IL-12 production in DCs and thus inducing a T_H_2 preferring milieu^23^. Furthermore it was reported, that pollen extracts contain substantial amounts of serine proteases^24^ or NAD(P)H oxidases^25^, both showing immunostimulatory effects.

Another potent cofactor in pollen extracts is lipopolysaccharide (LPS). It was reported that DCs are activated effectively through LPS recognition even at low doses, through a receptor complex consisting of toll-like receptor 4 (TLR4), CD14, and MD-2^26–29^. Because LPS is frequently present in birch pollen extracts as ubiquitous contaminant, which show a higher efficacy in SIT compared to the recombinant allergen, we aimed at studying the differences in the effects of a recombinant variant of Bet v 1.0101 (further referred to as Bet v 1), birch pollen extract (BPE), and pure LPS on human moDCs. We also investigated the combination of Bet v 1 and LPS in order to examine synergistic effects probably evoked by the endotoxin.

Immunological techniques such as enzyme linked immunosorbent assays (ELISA), fluorescence microscopy, and flow cytometry are powerful tools to investigate proteins of interest and their role in inflammation. However, to generate hypotheses regarding the underlying mechanisms in allergic immune responses and to identify determinants of allergenicity, a wholistic view on the complex interaction network of proteins in immunity is required. Shotgun proteomics is a suitable tool for more systemic investigations, overcoming the limitations of the techniques described above and providing a more comprehensive picture of the complexity of allergic immune responses^30^.

Here, we report the application of high-performance liquid chromatography (HPLC) and tandem mass spectrometry (MS/MS) for proteomic profiling of the allergic immune response in monocyte-derived dendritic cells (moDCs) which represent a broadly accepted *in vitro* model to study dendritic cell functions^31,32^. We found significant differences in the cellular response between treatments with Bet v 1 and BPE. In addition, our study suggests, that the effects observed upon BPE treatment clearly differ from LPS-induced responses. Furthermore our results show that a combination of Bet v 1 and LPS may induce additive but no synergistic effects. Thus, our findings confirm the assumption that the allergenicity of Bet v 1 is limited and that costimulatory factors including not only LPS are required to trigger an allergic immune response.

## Results and Discussion

### Dose finding experiments

**Figure 1:**
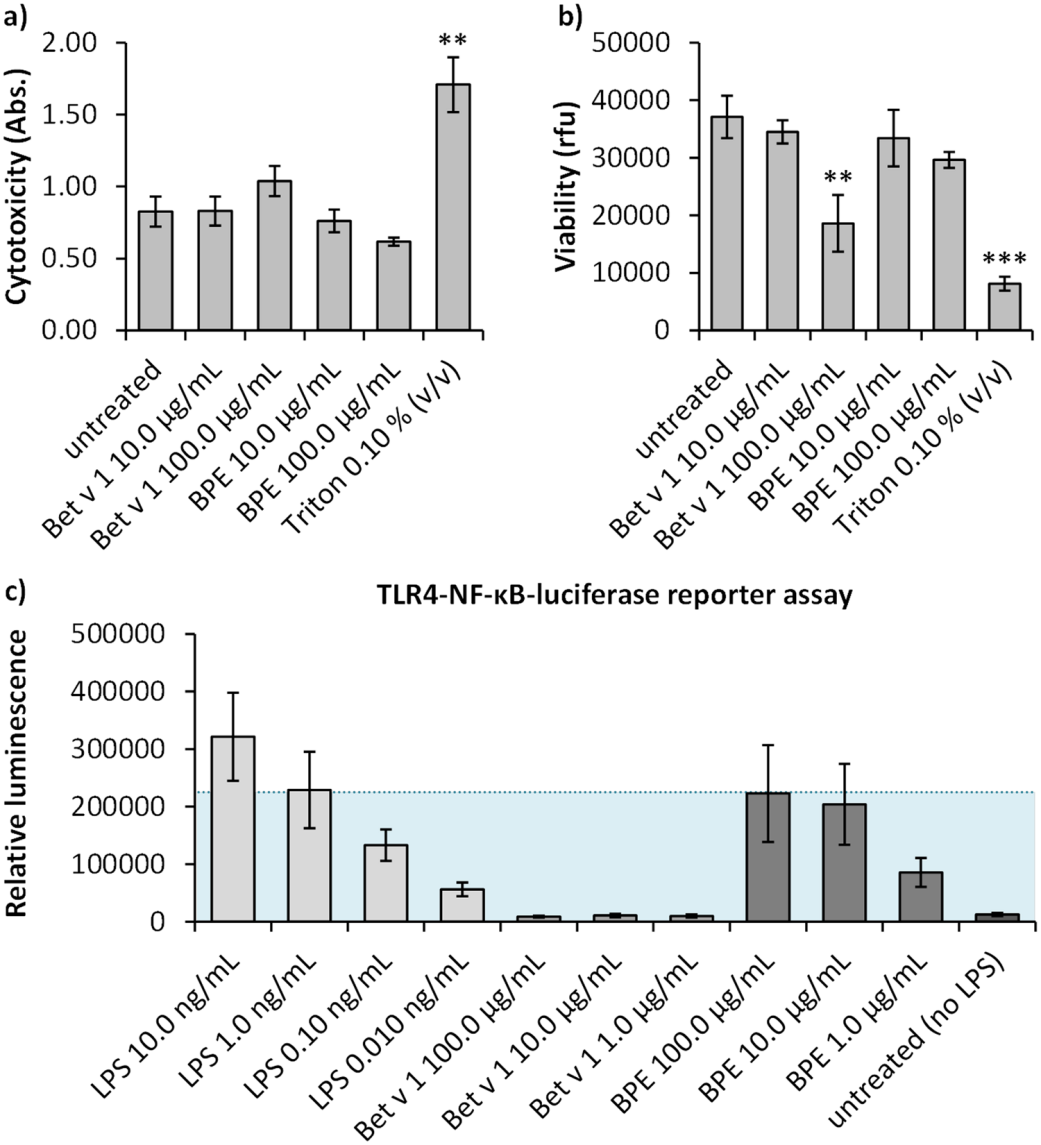
Cytotoxicity and cell viability of moDCs and NF-κB activation in HEK293 cells in response to Bet v 1, BPE and LPS. moDCs were treated with 10.0 and 100.0 µg/mL Bet v 1 or BPE for 24 h and assessed for (**a**) LDH release and (**b**) cell viability. Data represents the mean ± SD of three biological replicates. Statistical significance was determined by ANOVA with a Tukey’s post-test (*p ≤ 0.05; **p ≤ 0.01; ***p ≤ 0.001). Triton X-100 (0.10% v/v) was used as positive control for viability and cytotoxicity assay. (**c**) Activation of NF-κB in transfected HEK293 cells. Cells were treated with LPS (10.0–0.010 ng/mL), Bet v 1, and BPE (100.0–1.0 µg/mL). After treatment for 24 h luciferase activity was measured. Results show mean ± SD of two biological and two technical replicates. Dotted line shows that 10.0 µg/mL BPE equals to 1.0 ng/mL LPS.

To assess whether the used concentrations of Bet v 1 and BPE for stimulation during following experiments are toxic, a CellTiter-Blue^®^ (CTB) cell viability assay as well as a CytoTox 96^®^ Non-Radioactive Cytotoxicity assay were performed. As shown in Fig. 1a, there was no significant cytotoxicity of the used stimulants even at high doses. Fig. 1b shows the influence of the stimulants on viability of moDCs. Nevertheless, the highest concentration of Bet v 1 (100.0 µg/mL) significantly decreased viability of the cells. It could be speculated, that this is due to ligand binding of Bet v 1 which probably results in an increased cellular stress level^33^. Thus, 10.0 µg/mL of Bet v 1 and BPE were used for the following experiments.

To quantify LPS levels present in the used stimulants, TLR4-dependent NF-кB activation in transfected HEK293 cells was measured as displayed in Fig. 1c. None of the used concentrations of Bet v 1 induced a measurable increase in NF-кB activity, which leads to the conclusion that the used recombinant allergen was free of endotoxin contaminations. A concentration of 10.0 µg/mL BPE resulted in an NF-кB activation equivalent to 1.0 ng/mL LPS. Therefore, 1.0 ng/mL of LPS was used as reference for further experiments in order to distinguish between pure endotoxin-effects and BPE effects during proteome analysis of moDCs.

### Differential proteome analysis of moDCs after treatment with BPE, Bet v 1, and LPS

moDCs generated from nine individual donors (Fig. S1a) were stimulated with recombinant Bet v 1, BPE or LPS. After 8 hours, cells were harvested and cell lysates were pooled to reduce donor variability as depicted in Fig. S1b. Extracted and digested proteins were then separated by means of reversed-phase-HPLC followed by tandem MS-analysis on a hybrid quadrupole-Orbitrap mass spectrometer.

**Figure 2:**
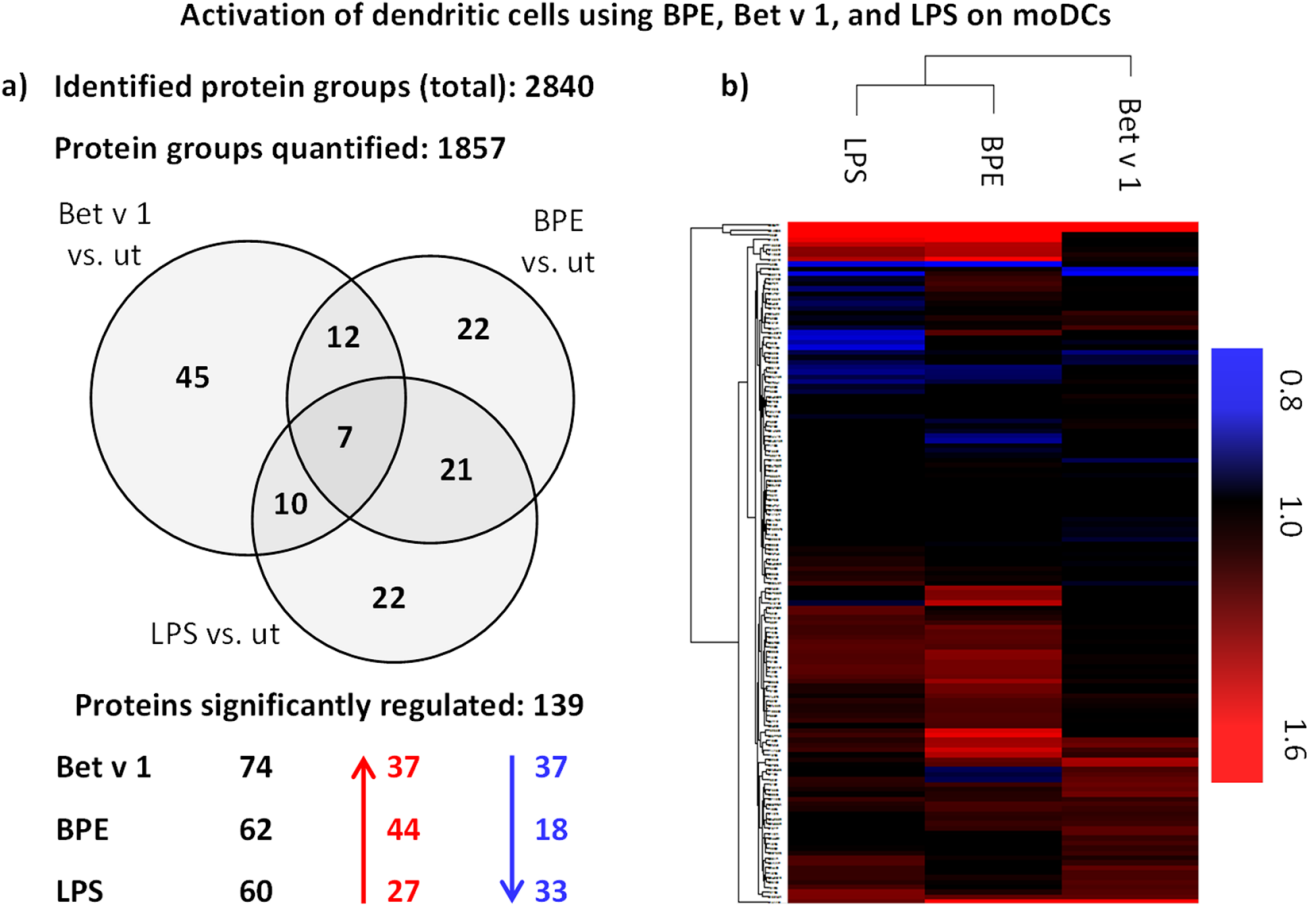
Quantified protein groups and significantly regulated proteins after treatment with Bet v 1, BPE, and LPS. (**a**) In total (moDCs obtained from 9 donors) 2840 protein groups were identified. 1867 protein groups were quantified. The Venn-diagram shows the overlap of significantly regulated protein groups (treated vs. untreated, “ut”) after the corresponding treatment. Proteins were considered to be significantly regulated if averaged and normalised ratios were greater or less than two times the standard deviation. (**b**) Hierarchical clustering based on Euclidian distance of 139 significantly regulated proteins after treatment of moDCs with Bet v 1, BPE, and LPS. Shown are iTRAQ^®^ ratios (treated *vs.* untreated, “ut”). Blue indicates downregulation, red highlights upregulation. The entire list of regulated proteins can be found in supplementary Excel sheet 1.

Mascot database search of obtained spectra using the Proteome Discoverer 1.4 software resulted in the identification of 2840 protein groups. Labelling with iTRAQ^®^ facilitated the quantification of 1857 protein groups. Ratios greater or less than two times the standard deviation of all measured ratios were considered to be significant (p < 0.023). Thereby 139 proteins were found to be significantly up- or downregulated. The entire list of identified and quantified protein groups can be found in worksheet 1 of the supplement (Excel sheet 1). Most significantly regulated protein groups were observed after Bet v 1 treatment (Fig. 2a). However, the overall extent of the regulation was much lower in comparison to BPE and LPS treatment. Comparison of regulated proteins after respective treatment in a Venn-diagram revealed an overlap of 5% (Fig. 2a). This low overlap leads to the assumption that the effects induced by the various treatments differ considerably. This finding is also confirmed by the heatmap illustrated in Fig. 2b.

**Figure 3:**
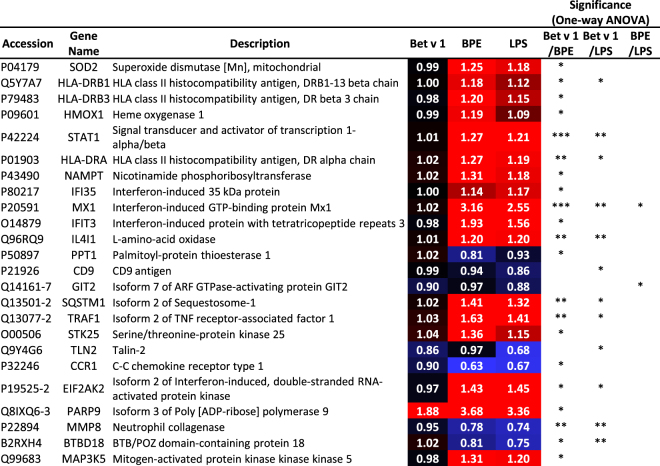
Regulated and differentially expressed proteins. 24 regulated proteins show significant differences in their expression when comparing Bet v 1, BPE, and LPS. Statistical evaluation was done using one-way ANOVA and a Tukey post-test. Shown are median ratios as well as significance (*p ≤ 0.05; **p ≤ 0.01; ***p ≤ 0.001) after comparison of treatments.

Fig. 2b gives an overview of the general regulation pattern considering the 139 proteins that were found to be significantly regulated. The entire list of significantly regulated proteins can be found in worksheet 2 of the supplement (Excel sheet 1). Some of those proteins show clear variances in the regulation when comparing the treatments. Since we were not only interested in the allergenicity of Bet v 1, but also aimed at a clear distinction between BPE- and LPS-induced effects, proteins differing in the respective expression were of special importance. By using one-way ANOVA we tested for significant differences between the employed treatments. Fig. 3 shows ratios (treated *vs*. untreated, represented as fold-changes) of 24 proteins, which were highly regulated as well as differentially expressed after treatment. Noteworthy, the intensity of regulation was generally lower after LPS treatment for almost every altered protein. The modest activation of DCs could be a consequence of the low LPS concentration used for these experiments, which was chosen based on the measured LPS contamination in BPE. Significant differences when comparing BPE and LPS were found for GIT2 (Q14161-7) and MX1 (P20591). According to gene ontology annotation, MX1 is involved in cytokine related signalling pathways. An upregulation after LPS treatment was already reported in literature which fits into our findings^34,35^. Therefore and because of the high response observed for MX1 (more than 3 times upregulated across BPE treatment) it becomes an interesting target for further analysis. GIT2, a GTPase-activating protein which was found to be downregulated after LPS treatment, is important for signal transduction^36^. So far, no immunological connection was described for this protein. Further substantial differences for proteins related to defence and immune system processes (*e.g*. HLA-DR3, HLA-DRA, STAT1, CCR1, and CD9) or oxidoreductases such as SOD2 were noticed when comparing Bet v 1 and BPE. As depicted in Fig. 3, those proteins were highly regulated after BPE treatment, whereas only poly (ADP-ribose) polymerase 9 (PARP9) was upregulated after Bet v 1 treatment as well. However, previous work mainly highlighted the role of PARP1 and PARP14 in allergic responses^37,38^. In line with this, it was recently published by Iwata *et al*. that PARP9 mainly promotes responses to IFNγ, whereas PARP14 suppresses IFNγ-induced responses and augments IL-4-responses, which rather highlights PARP14 than PARP9 as mediator of allergic responses^39^. Some of the other proteins mentioned in Fig. 3 were already reported to be involved in LPS response mechanisms such as NAMPT, IFI35, IFIT3, CD9, SQSTM1, and SOD2^35,40–42^. Especially the superoxide dismutase SOD2 seems to be of importance since it is needed for protection of cells against oxygen radicals. Oxygen radicals were described to be built in response to microbe infection^41,43^.

### Gene ontology enrichment analysis

**Figure 4:**
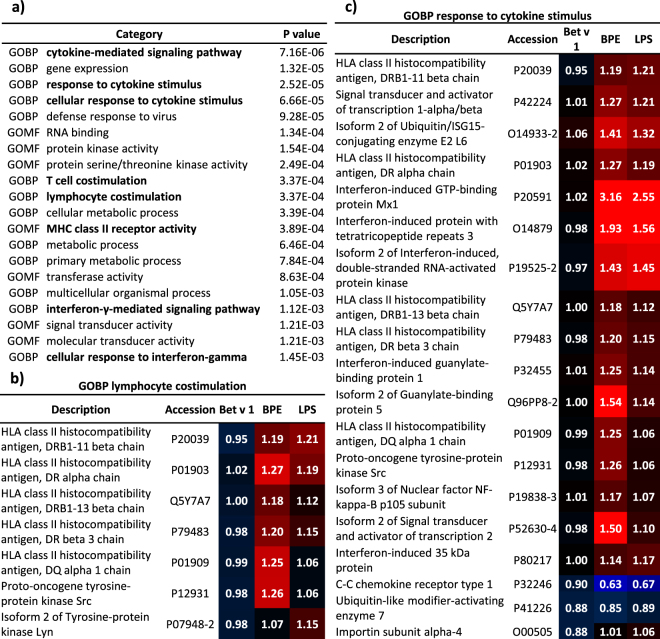
Gene ontology (GO) enrichment analysis. (**a**) Top 20 enriched gene ontology biological processes (GOBP) and molecular functions (GOMF) identified using significantly regulated proteins for Fisher’s exact test (p ≤ 0.01). Highlighted in bold are immunologically relevant processes. Heatmaps representing iTRAQ^®^ ratios of significantly regulated proteins connected to (**b**) lymphocyte costimulation, or (**c**) response to cytokine stimulus. Red indicates an upregulation and blue highlights downregulated proteins. Numbers in the heatmaps represent fold-changes relative to the untreated control.

On the basis of these findings, gene ontology enrichment analysis was performed to further elucidate the biological meaning of those regulated proteins. All regulated proteins were used for gene ontology (GO) enrichment analysis using a Fisher’s exact test with a p-value threshold of 0.01. As highlighted in Fig. 4a, cytokine related biological processes as well as MHC class II receptor activity and IFNγ related signalling pathways were enriched in our data. Additionally lymphocyte- and T cell costimulation were enriched. Furthermore, regulation of proteins related to gene expression, kinase activity as well as metabolic processes was observed.

Details on the expression of proteins related to biological processes such as response to lymphocyte costimulation and cytokine stimulus can be found in Fig. 4b,c. The heatmaps display ratios of regulated proteins linked to the particular function. There was no significant regulation of those proteins after the treatment with Bet v 1, whereas in some cases even a highly significant upregulation occurred after the treatment with BPE.

Moreover, several HLA class II histocompatibility antigens were found to be regulated after BPE and LPS treatment. These proteins are essential for MHC class II receptor activity and in further consequence also for lymphocyte costimulation (Fig. 4b). Furthermore, significant upregulation of proteins exclusively after the treatment with BPE, like Src (P12931) or HLA-DQA1 (P01909) was found, which makes them interesting for further analysis as well, just as MX1 and GIT2 already mentioned above.

A very similar pattern was detected for proteins participating in cytokine signalling (Fig. 4c). Here, the strong response after BPE treatment was visible for MX1 (P20591), IFIT3 (O14879) and EIF2AK2 (P19525-2). Once more a lower intensity of this response was detected after LPS treatment. There was no regulation observed for proteins mentioned above after treatment with Bet v 1, supporting our hypothesis that Bet v 1 is not affecting cellular processes important for the induction of an allergic immune response. More details on the expression of proteins involved in cellular processes and molecular functions mentioned in Fig. 4a as well as additional results of the GO enrichment analysis can be found in the supplement (Excel sheet 1 worksheet 3–23).

### Ingenuity pathway analysis

To further investigate as well as to differentiate the effects induced by pollen extract and LPS, the importance of regulated proteins in the context of different pathways was investigated by using QIAGEN’s Ingenuity Pathway Analysis.

Analysis of protein pattern changes revealed information about the activation or inhibition of canonical pathways. The predicted activation states of the most abundant pathways are depicted in supplementary Fig. S2 (activation is indicated by orange, blue shows inhibition according to activation z-score). In general, considerable differences in the activation states after various treatments were observed. Particularly the proteome analysis uncovered a significant activation of dendritic cell maturation, phospholipase C signalling, as well as calcium-induced T lymphocyte apoptosis and NRF2-mediated oxidative stress response after BPE treatment, whereas these pathways were at least not significantly affected by Bet v 1 or LPS. Furthermore, Fcγ receptor-mediated phagocytosis was predicted to be significantly inhibited after LPS treatment. Interestingly, Bet v 1 treatment resulted in an inhibition of ERK/MAPK signalling, as well as inhibited mTOR signalling. This shows that even though we did not observe a strong allergic response, moDCs are reacting to Bet v 1 present in the cell culture medium.

These observations fit into our previous findings. On the one hand, there was no significant activation of relevant pathways after Bet v 1 treatment indicating that Bet v 1 itself does not induce a strong immune response. On the other hand, differences in the level of significance when comparing effects of BPE and LPS were found, leading to the assumption that LPS is not the only important costimulatory factor in birch pollen.

### Investigation of potential synergistic effects of Bet v 1 and LPS using proteomics

**Figure 5:**
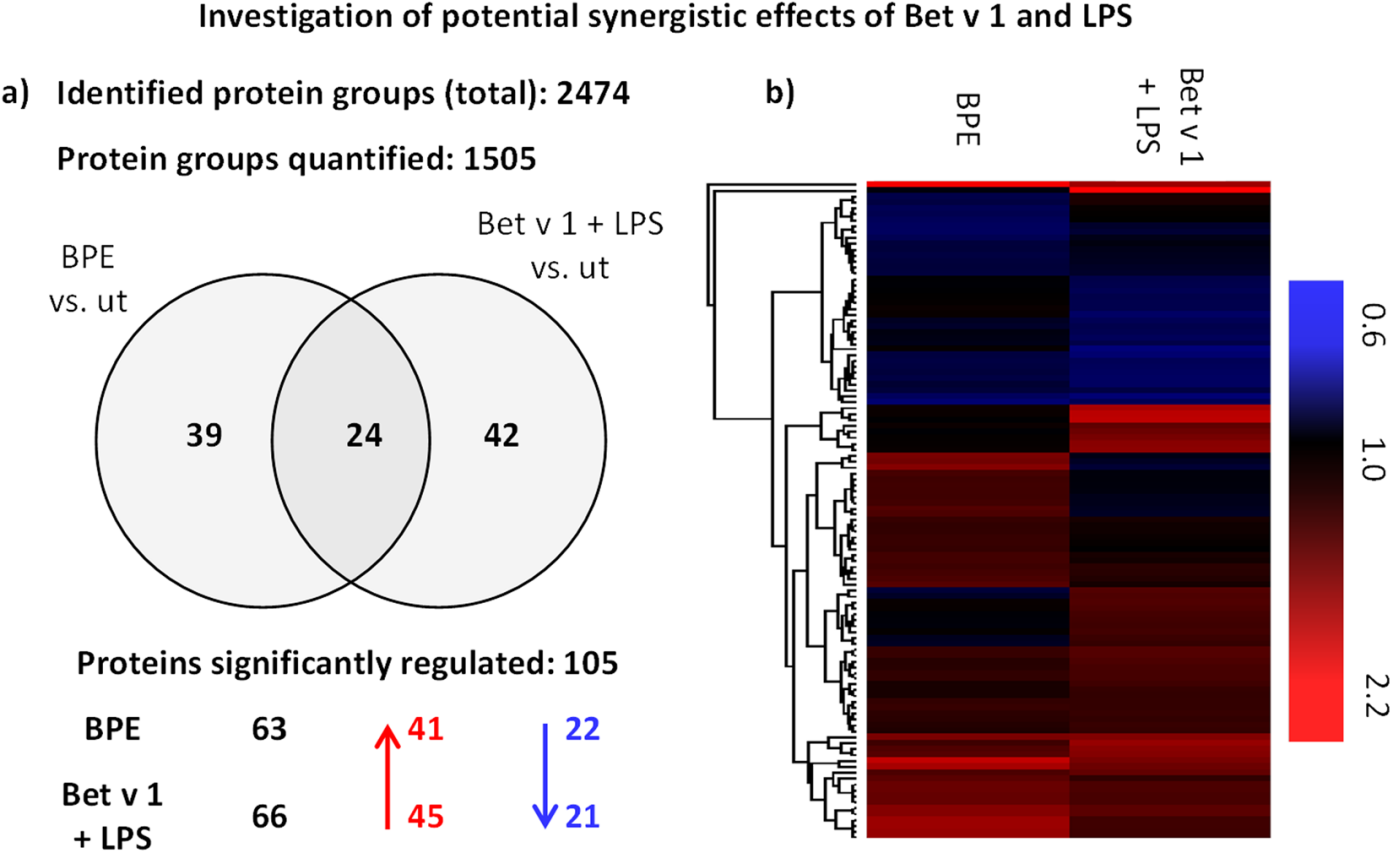
Quantified protein groups and significantly regulated proteins after treatment with BPE or Bet v 1 and LPS. (**a**) In total, 2474 protein groups were identified in moDCs obtained from 4 individual donors. 1505 protein groups were quantified. The Venn-diagram shows the overlap of significantly regulated protein groups (treated *vs.* untreated, “ut”) after the treatments. Proteins were considered to be significantly regulated if averaged and normalised ratios were greater or less than two times the standard deviation. (**b**) Hierarchical clustering based on Euclidian distance of 105 significantly regulated proteins after treatment of moDCs with BPE or Bet v 1 in combination with LPS. Shown are TMTsixplex™ ratios (treated *vs.* untreated, “ut”). Blue indicates downregulation, red highlights upregulation. The entire list of regulated proteins can be found in the supplementary excel file 2.

**Figure 6:**
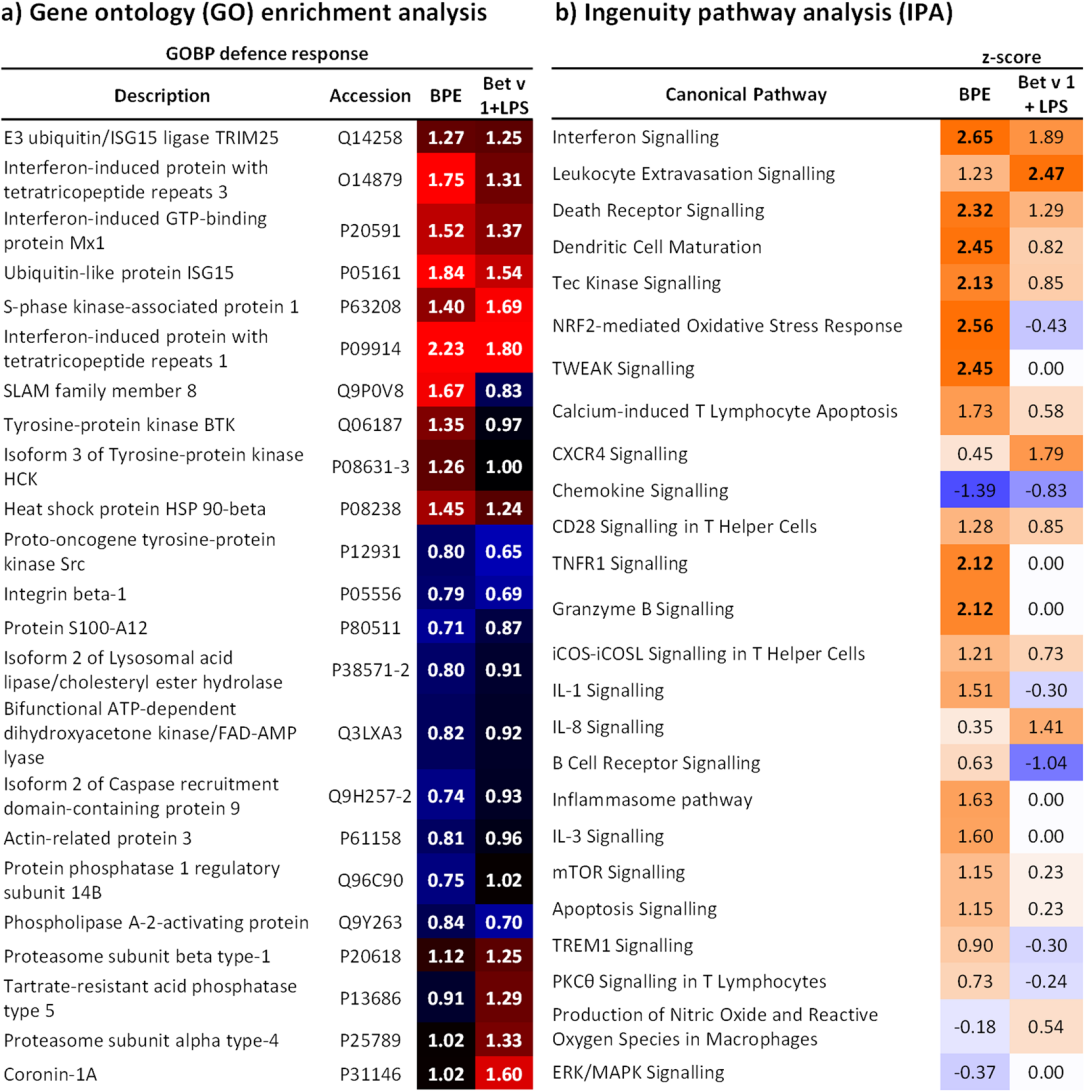
Gene ontology (GO) enrichment analysis and ingenuity pathway analysis (IPA) to examine potential synergistic effects of Bet v 1 and LPS. The heatmap in (**a**) is representing TMTsixplex™ ratios of significantly regulated proteins related to defence response mechanisms. Red indicates an upregulation and blue highlights downregulated proteins. Additionally predicted activation (orange) or inhibition (blue) of canonical pathways is shown in (**b**). Z-Scores greater ± 2.0 are considered to be significant.

In order to study whether DC maturation induced by BPE, can be fully mimicked by treatment with Bet v 1 and LPS, moDCs obtained from four individual donors were stimulated with BPE or a combination of Bet v 1 and LPS. For this treatment, similar concentrations of the allergen and LPS as measured in the BPE were used (Fig. S1c). As shown in Fig. S1d, cells were harvested after 8 hours of treatment for proteome analysis. As depicted in Fig. 5a, 2474 protein groups were identified and 105 were found to be significantly regulated. The entire list of identified and significantly regulated proteins can be found in the supplement (Excel sheet 2). When comparing the regulation pattern of those 105 proteins in a heatmap (Fig. 5b) distinct differences between stimulation with Bet v 1 + LPS and BPE stimulation are noticeable, indicating that the observed effects could be clearly distinguished. Thus, GO enrichment analysis and ingenuity pathway analysis were performed (Fig. 6).

By doing so, cytokine related biological processes and immune effector processes were found to be enriched in our data. Details on the regulation of proteins involved in defence mechanisms are shown in Fig. 6a. Noticeably, the regulation of proteins involved into defence response differs when comparing the effects of BPE with Bet v 1 and LPS. In general, BPE treatment was more intense except for 5 proteins like for example Coronin-1A. Interestingly, the main function of Coronin-1A is the regulation of cytoskeleton organization due to its actin-binding capacity. However, according to GO annotation it is also involved into immune responses such as the cellular response to IL-4 or T cell proliferation. Most importantly, we were able to confirm findings from previous experiments (activation of dendritic cells using birch pollen extract, Bet v 1, or LPS). IFIT3 (O14879) and MX1 (P20591) were again found to be significantly upregulated upon our treatment. Additionally, the regulation pattern was similar to the first experiment meaning that the effect of combining Bet v 1 with LPS induced a similar response as pure LPS. Furthermore, this observation consolidates the statement that MX1 and IFIT3 could be important targets for further analysis. Another interesting finding is the regulation of SLAMF8 (SLAM family member 8, Q9P0V8). Whereas BPE induced an upregulation of SLAMF8, Bet v 1 and LPS resulted in a downregulation of this protein. According to Kingsbury *et al*. SLAM proteins are important for B-cell signalling^44^ as well as important for T-cell activation^45^.

Ingenuity pathway analysis (Fig. 6b) showed that BPE treatment induced significant activation of different processes, including interferon signalling, dendritic cell maturation, or NRF2-mediated oxidative stress response. The predicted activation of those pathways also fits into our previous findings. Additionally, Bet v 1 and LPS once more were not resulting in a significant activation of mentioned pathways.

In summary, the observed results of this proteome analysis confirmed our assumption that there may be additive but no synergistic effects of Bet v 1 and LPS, indicating that the strong activation of moDCs observed upon BPE treatment might be explained by the presence of additional, so far not identified pollen-derived mediators. Additionally we were able to approve our previous findings such as the upregulation of MX1 or IFIT3 as well as an activation of dendritic cell maturation and NRF2-mediated oxidative stress response due to BPE treatment of moDCs.

### Treatment-associated cytokine release

**Figure 7:**
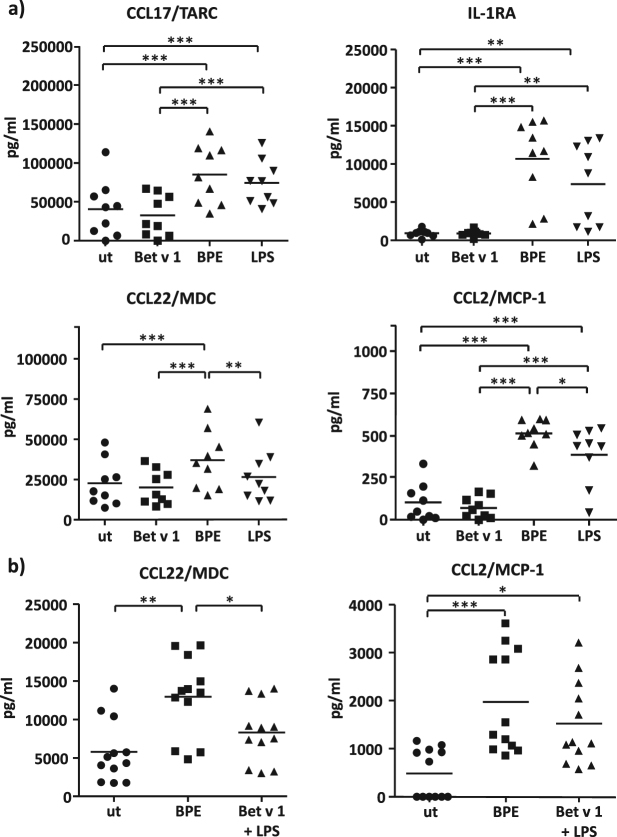
Effects of treatments on cytokine production in human moDCs. (**a**) 5 × 10^5^ moDCs/mL were stimulated as indicated (ut, Bet v 1, BPE, and LPS). After 8 h of incubation, supernatants of nine individual donors were harvested and analysed via ELISA (CCL17/TARC and CCL22/MDC) or multiplex assay (IL-1RA and CCL2/MCP-1). (**b**) 5 × 10^5^ moDCs/mL obtained from 4 individual donors were stimulated as indicated (ut, BPE, and Bet v 1 + LPS). After 8 h of incubation, supernatants were harvested and CCL22/MDC and CCL2/MCP-1 production was measured via ELISA (3 technical replicates). Bars represent mean values. For statistical analysis, ANOVA with a Tukey’s post-test was performed. *p ≤ 0.05; **p ≤ 0.01; ***p ≤ 0.001.

To corroborate our findings, we additionally applied well established immunological techniques. Thus, the treatment-dependent release of DC-derived cytokines and chemokines was measured by means of ELISA and Multiplex technology. Whereas the production of T_H_1 associated factors including IL-12 and IL-6 does not differ between LPS and BPE treated moDCs (Fig. S3), significant differences can be observed for T_H_2 related factors. Fig. 7a shows the concentration of CCL17/TARC, IL-1RA, CCL22/MDC, and CCL2/MCP-1 measured in supernatants of moDCs used for proteome analysis. As depicted, a highly significant increase for all cytokines was measured when treating the cells with BPE. Interestingly, all cytokines tend to be lower expressed upon LPS stimulation. For CCL22/MDC as well as CCL2/MCP-1 even a significant difference in the expression level was observed. CCL22/MDC and CCL17/TARC are expressed by antigen-presenting cells and take part in the T_H_2 amplification loop, by inducing the recruitment of IL-4 secreting T_H_2 cells^46^. CCL2/MCP-1 is believed to support the secretion of T_H_2 cytokines^47^ on top of it being an important T_H_2-attracting chemokine like CCL22/MDC and CCL17/TARC, indicating a similar importance for the outcome of a T_H_2 phenotype^48^. Thus, significantly higher CCL22/MDC and CCL2/MCP-1 production after BPE stimulation in comparison to LPS treatment further indicates differences in the resulting immune response. In contrast to LPS and BPE stimulation, there was no significant increase of T_H_2 related cytokines after stimulation with Bet v 1. This finding once more confirms the assumption that the allergen itself is not able to induce a strong immune response, whereas BPE and LPS treatment lead to a sufficient activation of the cells.

Similar effects were also reported when treating moDCs with a combination of Bet v 1 and LPS. As shown in Fig. 7b, both, CCL22/MDC as well as CCL2/MCP-1 were significantly upregulated after BPE treatment whereas the effect was distinctly lower after treatment with Bet v 1 and LPS, suggesting that the combination of the latter is less efficient in activating DC responses compared to BPE treatment.

### Expression of cell surface markers

**Figure 8:**
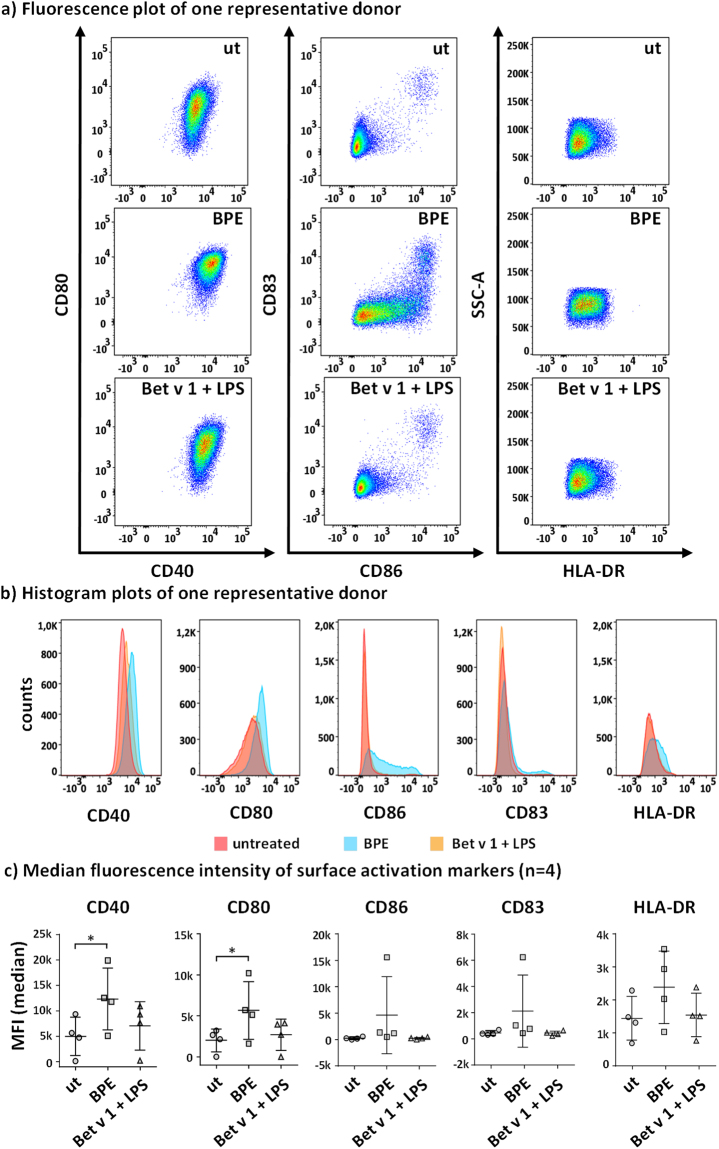
Flow cytometric analysis of surface activation markers on human moDCs stimulated with BPE and Bet v 1 + LPS. 5 × 10^5^ moDCs/mL were stimulated with 10.0 µg/mL BPE and 2 µg/mL Bet v 1 + 1 ng/mL LPS, which is the similar concentration found in the used BPE, respectively. After 24 h of incubation, cells were stained for the expression of CD40, CD80, CD83, CD86 and HLA-Dr and subsequent analysed by flow cytometry. (**a**) Fluorescence plot of one representative donor. Shown are CD80/CD40 and CD83/CD86 for untreated (ut) moDCs as well as stimulated cells. FACS histograms in (**b**) show expression of surface markers after BPE treatment (blue) or Bet v 1 + LPS (yellow) in comparison with ut (red) from the similar donor. In (**c**) median fluorescence intensity values of moDCs obtained from 4 donors are shown. For statistical analysis, ANOVA with a Tukey post-test was performed. *p ≤ 0.05.

Finally, effects of a combination of Bet v 1 and LPS were also investigated at the level of surface marker expression. Thus, for flow cytometric analysis 5.0 × 10^5^ cells/mL were seeded into 96-well plates (130 µL/well) and treated with BPE (10.0 µg/mL) or Bet v 1 (2.1 µg/mL) + LPS (1.0 ng/mL) for 24 h (Fig. S1e). Thereafter, cells were harvested and stained for the expression of CD40, CD80, CD83, CD86, and HLA-DR. As depicted in Fig. S4, approximately 80% of obtained cells are CD1a^+^ and no impaired viability was observed. Fig. 8a shows fluorescence plots of one representative donor. BPE-treatment resulted in upregulation of all tested surface markers, whereas the combination of Bet v 1 and LPS had no significant effect. Observed differences are conveyed in more detail in the histogram plots shown in Fig. 8b. Taking all 4 donors into accordance, especially CD40 and CD80 were significantly upregulated after BPE treatment, whereas no effects were observed upon stimulation with Bet v 1 and LPS (Fig. 8c).

## Conclusion

In this study, we used HPLC-MS-based proteome analysis to compare the effects of the major birch pollen allergen Bet v 1 and BPE in order to elucidate the allergenicity of Bet v 1. We reported a significantly lower intensity of response when treating moDCs with Bet v 1 compared to a strong reaction after treating cells with BPE. BPE significantly regulated the expression of proteins involved in various pathways including cytokine signalling as well as dendritic cell maturation whereas this was not the case for Bet v 1. However, since dendritic cells are highly sensitive to LPS, which is a known contaminant in pollen, we also discriminated the effects of LPS from those induced by BPE by comparing moDC responses upon LPS + Bet v1 and BPE stimulation. By doing so, we were able to record differences in effects induced by BPE from effects caused by ubiquitous endotoxin contaminations. In particular, we found proteins such as IFIT3 and MX1 to be differentially expressed upon the applied treatments, making them promising targets for further investigations.

Additionally, we were able to show that Bet v 1 and LPS may induce additive effects in moDCs but that the combination thereof is still not as efficient as treatment with BPE. Our findings on the level of protein expression have been additionally confirmed by measuring secretion of chemotactic cytokines (especially CCL22/MDC and CCL2/MCP-1) as well as the expression of surface markers (*e.g*. CD40 and CD80). According to these results we consider Bet v 1 alone not to be able to induce an allergic immune response. Furthermore we showed that LPS is not necessarily the sole factor needed to induce a T_H_2 biased immune response but other context-dependent mediators derived from the pollen may as well be essential in this process.

## Materials and Methods

All studies involving human cells were conducted in accordance with the guidelines of the World Medical Association’s Declaration of Helsinki. In this study, we used monocytic cells derived from human buffy coats. Our national regulations do not require informed consent in the case of anonymous blood cells discarded after plasmapheresis (buffy coats), therefore no additional approval by the local ethics committee is required. According to university guidelines, the project was approved by the head of the Department of Molecular Biology, University of Salzburg, Austria.

### Generation of human monocyte derived dendritic cells (moDCs)

moDCs used in the present study were generated from buffy coats provided by the blood bank Salzburg, Austria as previously published^32^. Briefly, peripheral blood mononuclear cells (PBMCs) of healthy donors were isolated after gradient centrifugation using Ficoll-Paque PLUS (GE Healthcare, Chalfont St Giles, U.K.). Remaining erythrocytes were lysed with ACK lysis buffer (150.0 mM ammonium chloride, 10.0 mM potassium bicarbonate, 0.10 mM EDTA obtained from MERCK, Darmstadt, Germany and VWR International, Radnor, PA, USA) followed by extensive washing with Gibco^®^ DPBS (Life Technologies, Carlsbad, CA, USA). Afterwards cells were diluted in DC-medium (RPMI 1640 medium containing 10.0% heat-inactivated foetal calf serum (FCS), 100.0 U/mL penicillin, 100.0 µg/mL streptomycin, 2.0 mM L-glutamine and 50.0 µM 2-mercaptoethanol from Sigma Aldrich, St. Louis, MO, USA) to achieve 1.0 × 10^7^ cells/mL. PBMCs were left to adhere in six-well plates for 70 min at 37.0 °C and 5.0% CO_2_. Subsequently non-adherent cells were removed by washing with pre-warmed RPMI 1640 medium. Monocytes were then stimulated with DC-medium containing 50.0 ng/mL GM-CSF and 50.0 ng/mL IL-4 (PeproTech, Rocky Hill, NJ, USA) for six days. On day three of cultivation one volume of DC medium with 100.0 ng/mL cytokines was added in order to generate moDCs.

### Stimulants

*E. coli* lipopolysaccharide (LPS) 055:B5 was obtained from Sigma-Aldrich, St. Louis, MO, USA. Recombinant Bet v 1.0101 (Bet v 1) and birch pollen extract (BPE, containing 20.08% Bet v 1) were kindly provided by Claudia Asam from the working group of Fátima Ferreira, University of Salzburg, Austria. The LPS content within the recombinant protein as well as in the pollen extract was determined by luciferase assay. The Bet v 1 concentration in BPE was determined by Lorenz Aglas (working group of Fátima Ferreira) using ELISA. Exposure times as well as the concentration were dependent on the assay.

### Luciferase assay

HEK293 cells were cultivated in DMEM medium (Sigma Aldrich, containing 10.0% FCS, 2.0 mM L-glutamine, 2.0 mM MEM non-essential amino acids, 100.0 U/mL penicillin, 100.0 µg/mL streptomycin). For the luciferase assay 1.5 × 10^5^ cells per well were plated in 24-well plates and transfected by using Lipofectamine 2000 reagent (Invitrogen, Life Technologies). As published by Schwarz *et al*.^26^, a reaction solution was prepared consisting of 400.0 ng NF-кB luciferase reporter with a TLR4-CD14-MD2 mix (ratio 3:1:1; NF- кB:TLR4 mix ratio 4:1) diluted in 1.50 µL Lipofectamine 2000 reagent and 50.0 µL Opti-MEM (Gibco). After five min of incubation, the reaction solution was added to the wells. After cells were transfected for 24 h, the medium was replaced by fresh medium and cells were treated with Bet v 1 or BPE of different concentrations (1.0–100.0 µg/mL) for another 24 hours. After induction, supernatants were discarded and 100.0 µL lysis buffer (100.0 mM potassium phosphate (Carl Roth GmbH + Co. KG., Karlsruhe, Germany), 0.10% Triton X-100 (Bio-Rad laboratories, Richmond, California), 1.0 mM DTT (Sigma Aldrich), pH 7.8) were added to each well. Cell lysates were transferred into white polystyrene flat-bottom microtiter plates and luciferase activity was determined by using a Tecan Infinite 200 Pro microplate reader (Tecan, Groedig, Austria) after the addition of 50.0 µL luciferase substrate.

### Cytotoxicity and cell viability

moDCs were seeded into a 24-well plate at a concentration of 1.0 × 10^5^ cells/mL and treated with the respective stimuli for 24 h, as recommended by the assay protocol. Cytotoxicity was assessed by determining the lactate dehydrogenase (LDH) content in cell supernatants by using the CytoTox 96^®^ Non-Radioactive Cytotoxicity assay from Promega (Madison, WI, USA). As a control for maximum LDH release, cells were treated with 0.10% (v/v) Triton X-100 for 30 min. After induction supernatants were transferred into 96-well plates and according to the manufacturer mixed with CytoTox 96^®^ reagent. After incubation for one hour at 37 °C LDH-substrate was added and cells were incubated for another 30 min at room temperature under protection from light. Subsequently the provided stop-solution was added and absorbance was measured at 492 nm by using a microplate reader (Tecan).

The influence of used stimulants on cell viability was determined by performing the CellTiter-Blue^®^ (CTB) cell viability assay (Promega). Again, Triton X-100 (0.10%) was used as a positive control. The remaining cells were mixed with the CTB-reagent in a ratio of 1:5 and incubated for one hour at 37 °C in the dark. Afterwards cells were pelleted, supernatants were transferred into a 96-well plate and fluorescence intensity was measured at ex/em wavelengths of 560 nm/590 nm.

### moDC stimulation and sample preparation for proteome analysis

For proteome analysis, moDCs were generated from nine healthy donors. Cells of each donor were seeded into 6-well plates at a concentration of 5.0 × 10^5^ cells/mL. Cells were then stimulated with 10.0 µg/mL Bet v 1, 10.0 µg/mL BPE or 1.0 ng/mL LPS for 8.0 h (Fig. S1b). A treatment period of 8.0 h was chosen in order to allow enough time for evolution of a proteomic response while avoiding secretion of relevant proteins that would escape intracellular proteome analysis. Subsequently, moDCs were harvested by centrifugation at 1,100 rpm for 10 min. Supernatants were collected and stored at −20 °C for further analysis. Cell pellets were washed with DPBS. Afterwards cells were dissolved in DPBS containing protease inhibitor (protease inhibitor cocktail tablets, cOmplete, Mini, EDTA-free, Roche, Basel, Switzerland) followed by sonication with an in-probe-sonicator from Branson Ultrasonics Corporation (Danbury, CT, USA) for 20 s on ice. For denaturation 50.0% (v/v) 2,2,2-trifluoroethanol (TFE; Sigma Aldrich) was added and the samples were incubated for 60 min at 60.0 °C on a Thermomixer Comfort (Eppendorf, Hamburg, Germany). For reduction of disulphide bonds, tris(2-carboxyethyl)phosphine hydrochloride solution (TCEP; Sigma Aldrich) was added to reach a final concentration of 4.55 mM followed by incubation at 60.0 °C for 60 min. Prior to alkylation, samples were cooled down to room temperature followed by the addition of iodoacetamide (IAA; Sigma Aldrich) to a final concentration of 8.70 mM. The samples were then incubated for 30 min at room temperature under protection from light. After alkylation proteins were precipitated with ice-cold acetone (Sigma Aldrich) at −20 °C overnight.

Proteins were then pelleted by centrifugation at 14,000 rpm for 50 seconds. Supernatants were discarded and protein pellets were dissolved in 0.10 M triethylammonium bicarbonate buffer (TEAB; Sigma Aldrich). Protein concentration was determined using the Pierce™ 660 nm protein assay kit (Thermo Fisher Scientific, Waltham, MA, USA). Proteins were digested with sequencing grade modified trypsin (Promega) and labelled with isobaric tags for relative and absolute quantitation (iTRAQ^®^, Sigma Aldrich) according to the manufacturer’s protocols. In order to minimize donor variability, peptides (10 µg per sample) obtained from three donors were pooled in a random fashion prior to the iTRAQ^®^ labelling resulting in pools representing three biological replicates. Samples were then analysed by high performance liquid chromatography-mass spectrometry (HPLC-MS).

### Investigation of potential synergistic effects of Bet v 1 and LPS using HPLC-MS

On behalf of a follow-up experiment, moDCs were generated from four healthy donors. Cells were seeded into 6-well plates at a concentration of 5.0 × 10^5^ cells/mL for proteome analysis followed by treatment with 10.0 µg/mL BPE or a combination of Bet v 1 (2.1 µg/mL) and LPS (1.0 ng/mL) for 8 h (Fig. S1c). Protein extraction as well as digestion was performed as described above. For relative quantitation (Fig. S1d), tryptic peptides were labelled using TMTsixplex™ Isobaric Label Reagent Set (Thermo Fisher Scientific) according to the manufacturer’s protocol, followed by HLPC-MS analysis.

### High-Performance Liquid Chromatography-Mass Spectrometry (HPLC-MS)

Labelled peptides were dissolved in 0.10% (v/v) formic acid (FA; Sigma Aldrich) in a concentration of 2.0 µg/µL. By using a 300 nL μL-Pickup injection, peptides were injected into an UltiMate^®^ 3000 RSLCnano HPLC system (Thermo Scientific). A self-packed 200 × 0.1 mm i.d. Hypersil GOLD™ aQ C18 capillary column packed with 3.0 µm particles was used for separation. Water (A) and acetonitrile (B; Sigma Aldrich) each containing 0.10% (v/v) FA were used as eluents. At a flow rate of 350 nL/min peptides were separated by applying a linear gradient of 5.0–40.0% B in 300 min. The column temperature was set to 50 °C. The HPLC system was on-line hyphenated to a Q Exactive™ Plus Hybrid quadrupole-Orbitrap™ mass spectrometer (Thermo Scientific) by means of a nano-electrospray ionization source. The source was operated in positive ionization mode with a spray voltage of 1.6 kV. Full scans were performed at a scan range of *m/z* 450 to 2,000 at a resolution of 70,000 (at *m/z* = 200). The AGC-target was set to 1 × 10^6^ with a maximum injection time (IT) of 100 ms. Data dependent MS^2^ spectra for the 10 most abundant ions were achieved by HCD fragmentation at 29% normalised collision energy at a resolution of 35,000 (at *m/z* = 200) followed by dynamic exclusion of ions already isolated for fragmentation for a time window of 15.0 seconds. For MS^2^ scans a fixed first mass was set to *m/z* 100, the AGC target was 1 × 10^5^ with a maximum IT of 60 ms. The used underfill ratio was 1.0% resulting in an intensity threshold of 1.7 × 10^4^. Analysis of TMT-labelled peptides was performed using a Q Exactive™ Hybrid Quadrupole-Orbitrap™ Mass Spectrometer (Thermo Scientific) with only minor changes in the method setup which are described in detail in the supplementary section.

### Data analysis

The mass spectrometry proteomics data have been deposited to the ProteomeXchange Consortium via the PRIDE^49^ partner repository with the dataset identifier PXD006196. For protein identification, the MS/MS spectra were searched against a human UniProt sequence database (released Jan 22, 2014, selected for *Homo sapiens*, 39 754 entries) using Proteome Discoverer 1.4 (Thermo Fisher Scientific). For MS spectra a mass tolerance of 10 ppm and 0.3 Da for MS/MS spectra as well as a maximum of one missed cleavage were allowed. Additionally, search criteria included a carbamidomethylation on cysteines and iTRAQ^®^ 4-plex labelled peptide amino terminus/lysine (+144.102 Da) or TMTsixplex™ (+229.163 Da) respectively as fixed modification as well as methionine oxidation and deamidated asparagine/glutamine as variable modifications. The FDR was set to <0.01.

Protein identifications obtained from Proteome Discoverer 1.4 were further analysed using Perseus (version 1.5). Log2(x)-ratios obtained from three biological replicates were normalised on the most frequent value followed by averaging replicates (median). Ratios greater or less than two times the standard deviation were considered to be significant. Furthermore a gene ontology (GO) enrichment analysis was performed based on GO molecular functions (GOMF) and GO biological processes (GOBP). Additionally proteins were imported into the QIAGEN’s Ingenuity^®^ Pathway Analysis™ software (IPA^®^, QIAGEN Redwood City, www.ingenuity.com) to reveal protein and interactive pathway regulation in response to various treatments.

### Enzyme-linked immunosorbent assay (ELISA)

Human CCL17/TARC and CCL22/MDC DuoSet ELISAs (cat. no. DY364 and DY336) were purchased from R&D Systems (Bio-Techne Ltd., Abingdon, UK). Human MCP-1 Mini ABTS ELISA Development Kit was obtained from PeproTech. Capture antibodies were coated on NUNC MaxiSorp flat-bottom 96-well plates (eBioscience, Affymetrix, Vienna, Austria) o.n. at 4 °C. Blocking was performed for 1 h at RT using DPBS supplemented with 1% BSA. Supernatants were added and incubated for 2 h at RT. Biotinylated detection antibodies were added for 1 h at RT. Avidin-conjugated horseradish peroxidase was added for another 30 min. After each step, plates were washed using DPBS containing 0.05% Tween-20. Then, 3,3′,5,5′-Tetramethylbenzidine substrate (Sigma-Aldrich, Vienna, Austria) was added and the reaction was stopped by 2 M sulfuric acid. Colour intensity was measured at 450 nm and a reference wavelength of 650 nm was subtracted. The accompanying standards were used to calculate total protein concentrations.

### Multiplex assay

IL-1RA and CCL2/MCP-1 beads were part of the ProcartaPlex Human Cytokine & Chemokine Panel 1A (34 plex) (eBioscience) which was used based on a modified protocol. Briefly, lyophilized standards were reconstituted in 250 µL DC-medium per vial. 8.34 µL of each bead mix were pooled per standard or sample, washed twice (5′ at 3000 × g) with wash buffer (DPBS containing 0.05% Tween-20) and re-suspended in assay buffer (DPBS containing 0.05% Tween-20 and 1% heat-inactivated FCS). 15 µL of standard or sample were added to 8.34 µL beads in a 96-well v-bottom plate and incubated o.n. at 4 °C in the dark on an orbital shaker set to 450–600 rpm. After o.n. incubation, the plate was washed thrice using wash buffer. For this, 130 µL wash buffer was added to each well and the plate was centrifuged at 1550 × g for 4 min. Supernatants were discarded by inverting the plate. This step was repeated two more times. After washing, beads were re-suspended in 15 µL of detection antibody (diluted 1:128 in assay buffer) and incubated for 1 h at RT on an orbital shaker. Thereafter, the plate was washed again three times and the beads were re-suspended in 20 µL Streptavidin-PE solution (diluted 1:1 in assay buffer). After 30 min of incubation, beads were washed three more times and re-suspended in 100 µL of MAGPIX Drive Fluid. Measurement was performed on a Luminex MAGPIX instrument using Luminex xPONENT software. Data was analysed using Procarta Plex Analyst Software (eBioscience). GraphPad Prism 5 software was used for statistical analysis. Data are expressed as mean plus standard deviation (SD). To compare multiple groups for statistical differences a one-way ANOVA with Tukey’s post-hoc test was performed. P values of p < 0.05 were considered statistically significant. (*p < 0.05, **p < 0.01, ***p < 0.001).

### Flow cytometry

For flow cytometric analysis, 5 × 10^5^ moDCs/mL were treated with 10 µg/mL BPE and 2.1 µg/mL Bet v 1 + 1.0 ng/mL LPS respectively (Fig. S1e). After incubation for 24 h, cells were washed with ice-cold DPBS and stained in 100 μl DPBS containing fluorescent-labelled antibodies for 30 min under protection from light. After extensive washing, staining was fixed by adding 4% (v/v) paraformaldehyde (PFA, Sigma-Aldrich) for 10 min. Then, cells were admitted into 200 µL DPBS containing 3 mM EDTA. The median fluorescence intensity (MFI) of 1 × 10^4^ cells was recorded for every sample using the FACS Canto™ II flow cytometer operated with FACS Diva Software v8.0 (BD Biosciences, Vienna, Austria). Data analysis was performed using the FlowJo^®^ Software v10.3 (FlowJo LLC, Ashland, Oregon, USA).

EBioscience™ fixable Viability Dye eFluor® 506, anti-human CD3 eFluor® 506 (clone: UCHT1), anti-human CD19 eFluor® 506 (HIB19), anti-human CD40 FITC (5C3), anti-human CD86 PE (IT2.2), and anti-human HLA-DR APC (LN3) were purchased from Thermo Fisher Scientific. Brilliant Violet 421™ anti-human CD1a (Hl149) was obtained from BioLegend (San Diego, CA, USA). APC-H7 mouse anti-human CD80 (L307.4) and PE-Cy™7 mouse anti-human CD83 (HB15e) were purchased from BD Biosciences.

## Electronic supplementary material

Supplementary Information

Dataset 1

Dataset 2
