# Supplementary material for: Unbiased Quantitative Proteomics Reveals a Crucial Role of the Allergen Context for the Activation of Human Dendritic Cells
Source: Sci Rep. 2017 Nov 30;7:16638. doi: 10.1038/s41598-017-16726-2 (PMC5709417; doi:10.1038/s41598-017-16726-2)
Supplement: Supplementary file 1 — Supplementary Information [file 41598_2017_16726_MOESM1_ESM.doc]

**Unbiased Quantitative Proteomics Reveals a Crucial Role of the Allergen Context for the Activation of Human Dendritic Cells**

L. Strasser^1^, H.-H. Dang^2^, H. Schwarz^2^, C. Asam^2^, F. Ferreira^2^, J. Horejs-Hoeck^2^, C. G. Huber^1*^

1. Department of Molecular Biology, Division of Chemistry and Bioanalytics, University of Salzburg, Hellbrunner Straße 34, 5020 Salzburg, Austria

2. Department of Molecular Biology, Division of Allergy and Immunology, University of Salzburg, Hellbrunner Straße 34, 5020 Salzburg, Austria

**Corresponding author*

Christian G. Huber

Department of Molecular Biology, Division of Chemistry and Bioanalytics

University of Salzburg

Hellbrunner Straße 34

5020 Salzburg

Austria

Tel: +43 662 8044 5738

Fax: +43 662 8044 5751

E-mail: c.huber@sbg.ac.at

**Figure S1:** **Overview of the experimental design and workflow.** In order to investigate the capability of Bet v 1 to induce an allergic immune response, the focus of the first experiment was the discrimination of effects caused by Bet v 1.0101 (Bet v 1), birch pollen extract (BPE), and lipopolysaccharide (LPS). Thus (**a**) peripheral blood mononuclear cells (PBMCs) were isolated from human blood obtained from 9 individual donors followed by differentiation with interleukin 4 (IL-4) and granulocyte-macrophage colony-stimulating factor (GM-CSF) into monocyte derived dendritic cells (moDCs). (**b**) MoDCs obtained from 9 donors were then stimulated with recombinant Bet v 1, BPE, and LPS for 8.0 h, respectively. Stimulated moDCs were then harvested followed by protein extraction and tryptic digestion. Peptides obtained from 3 donors were pooled to reduce donor variability followed by iTRAQ® labelling. Labelled peptides were analysed using HPLC-MS/MS.

As to study synergistic effects probably evoked by a combination of Bet v 1 and LPS (lower part) moDCs obtained from additional 4 donors were treated with BPE or Bet v 1 and LPS at similar concentration shown to be present in the pollen extract (**c**). After eight hours (**d**) cells were harvested for differential proteome analysis using TMTsixplex™ labelling and supernatants were collected to measure cytokine production using ELISA. Additionally, cells were treated for 24 h (e) for flow cytometric analysis of surface activation markers.

**Figure S2: Ingenuity pathway analysis.** Heatmap displays activation z-scores of identified canonical pathways by using QIAGEN’s Ingenuity® Pathway Analysis™ software. Orange indicates a predicted activation whereas blue shows inhibition of the corresponding pathway. Z-Scores greater ± 2.0 are considered to be significant.

**
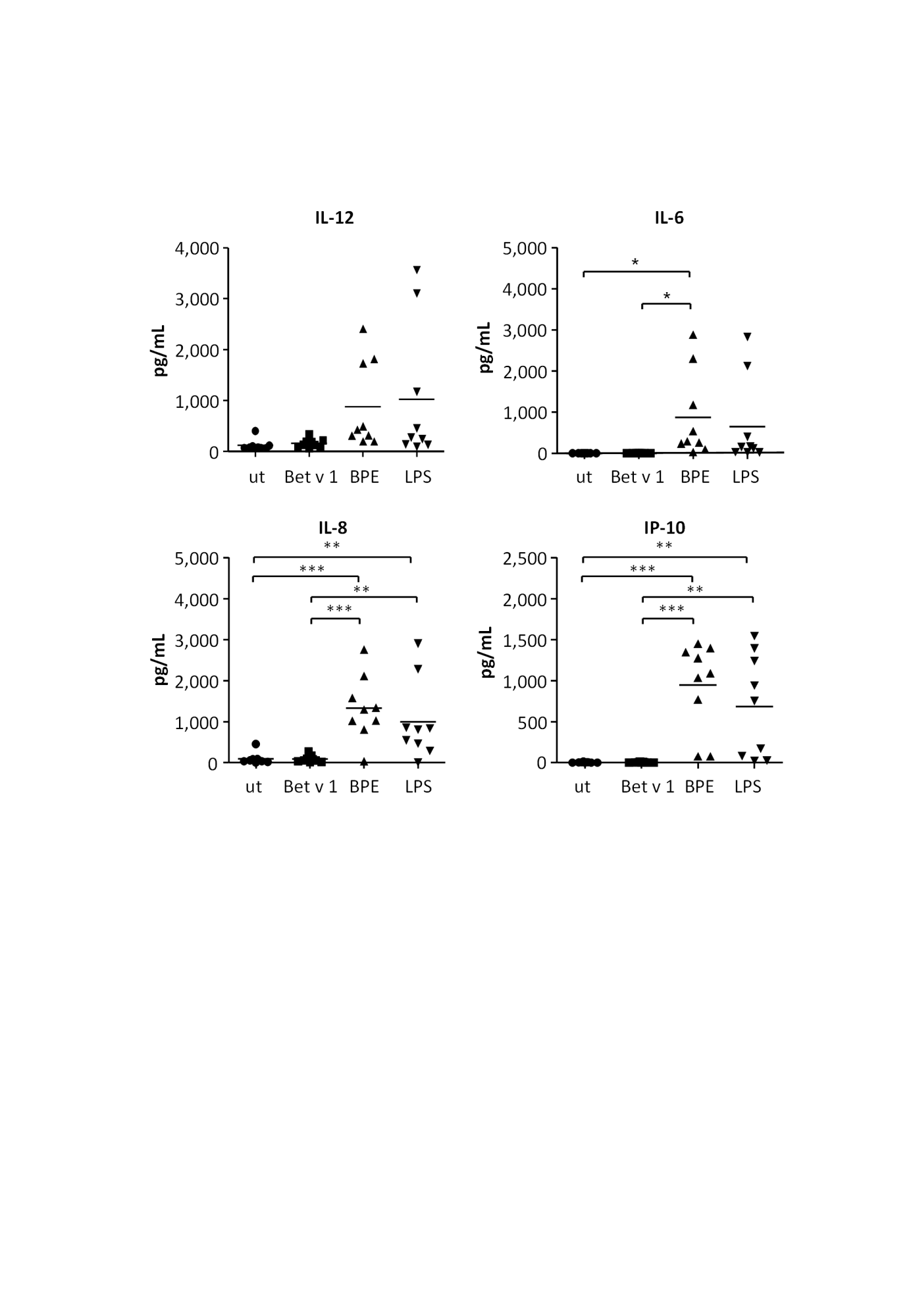
**

**Figure S****3: Effects of treatments on IL-12, IL-6, IL8, and IP-10 production in human moDCs.** 5 × 10^5^ moDCs/mL obtained from 9 individual donors were stimulated as indicated (ut, Bet v 1, BPE, and LPS). After 8 h of incubation, supernatants were harvested and analyzed via multiplex assay (IL-6, IL-8, and IP-10) or ELISA (IL-12). Bars represent mean values. For statistical analysis, ANOVA with a Tukey post-test was performed. * p ≤ 0.05.

**Figure S4: Flow cytometric analysis of human moDCs to check for purity and phenotype and viability of used cells.** 5 × 10^5^ moDCs/mL were stimulated with 10.0 µg/mL BPE and 2 µg/mL Bet v 1 + 1 ng/mL LPS, respectively. Viability and purity of moDC was determined by means of the EBioscience™ fixable Viability Dye eFluor® 506 (live/dead), which irreversibly stains dead cells, and by the analysis of CD1a expression, respectively.

**Materials and Methods**

**High-Performance Liquid Chromatography-Mass Spectrometry (HPLC-MS) for the investigation of synergistic effects of Bet v 1 and LPS using HPLC-MS**

Labelled peptides were dissolved in 0.10 % (v/v) formic acid (FA; Sigma Aldrich) in a concentration of 2.0 µg/µL. By using a 300 nL μL-Pickup injection, peptides were injected into an UltiMate® 3000 RSLCnano HPLC system (Thermo Scientific). A self-packed 200 x 0.1 mm i.d. Hypersil GOLD™ aQ C18 capillary column packed with 3.0 µm particles was used for separation. Water (A) and acetonitrile (B; Sigma Aldrich) each containing 0.10 % (v/v) FA were used as eluents. At a flow rate of 350 nL/min peptides were separated by applying a linear gradient of 5.0 – 40.0 % B in 300 min. The column temperature was set to 50 °C. The HPLC system was on-line hyphenated to a Q Exactive™ Hybrid Quadrupole-Orbitrap™ Mass Spectrometer (Thermo Scientific) by means of a nano-electrospray ionization source.

The source was operated in positive ionization mode with a spray voltage of 1.8 kV. Full scans were performed at a scan range of *m/z* 350 to 2,000 at a resolution of 70,000 (at *m/z* = 200). The AGC-target was set to 1x10^6^ with a maximum injection time (IT) of 120 ms. Data dependent MS^2^ spectra for the 15 most abundant ions were achieved by HCD fragmentation at 29 % normalised collision energy at a resolution of 35,000 (at *m/z* = 200) followed by dynamic exclusion of ions already isolated for fragmentation for a time window of 45.0 seconds. For MS^2^ scans a fixed first mass was set to *m/z* 110, the AGC target was 5x10^5^ with a maximum IT of 120 ms. The used underfill ratio was 2.5 % resulting in an intensity threshold of 1.0x10^5^.
